# 248例胸腔镜肺叶切除术手术心得

**DOI:** 10.3779/j.issn.1009-3419.2011.06.08

**Published:** 2011-06-20

**Authors:** 真榕 张, 德若 刘, 永庆 郭, 彬 石, 燕雏 田, 之已 宋, 海涛 张, 朝阳 梁

**Affiliations:** 100029 北京，中日友好医院胸外科 Department of Toracic Surgery, China-Japan Friendship Hospital, Beijing 100029, China

**Keywords:** 胸腔镜手术, 肺肿瘤, 并发症, VATS, Lung neoplasms, Complication

## Abstract

**背景与目的:**

早期肺癌胸腔镜肺叶切除术（video-assisted thoracic surgery, VATS）目前已经成为早期非小细胞肺癌（non-small cell lung cancer, NSCLC）治疗的常规术式。本文对中日友好医院胸外科已完成的胸腔镜肺叶切除术术中应急处理情况以及胸腔镜与传统开胸手术围手术期的相关因素进行分析总结。

**方法:**

对2006年1月-2008年7月在中日友好医院胸外科进行的肺癌手术患者进行回顾性研究。

**结果:**

共实施胸腔镜肺叶切除术248例，其中完全胸腔镜（complete video-assisted thoracic surgery, CVATS）组117例，胸腔镜辅助（assisted video-assisted thoracic surgery, AVATS）组131例。其中CVATS中转为AVATS或开放手术（open lobectomy, OPEN）共13例。中转术式的最常见原因依次为肺动脉或其分支出血、胸腔粘连、血管解剖变异、奇静脉出血、中叶静脉出血等。入组OPEN手术患者共129例。与OPEN组相比，VATS组的住院时间较短（20天*vs* 27天，*P*=0.015）、术中失血量较少（197 mL *vs* 250 mL, *P*=0.005），患者术后疼痛较轻（4.6 *vs* 6.2, *P*=0.003）。

**结论:**

胸腔镜手术具有风险小、安全性较高、患者恢复快等特点，因此在一定范围内可以代替传统开胸手术。

## Abstract (en)

**Background and objective:**

VATS-lobectomy has been used as regular surgical procedure clinically for non-small cell lung cancer. The aim of this study is to evaluate the surgical emergencies during VATS and the related factors postoperatively.

**Methods:**

Clinical data were reviewed for patients who were performed with pulmonary related surgery between January 2006 and July 2008 in our department.

**Results:**

248 (117 CVATS and 131 AVATS) VATS lobectomy were performed, including 13 cases that were transferred into AVATS or OPEN. The common related reasons were bleeding of pulmonary branches, adhesion, anatomic deformity, bleeding of azygos and bleeding of middle-lobe-vein. 129 thoracotomy cases were enrolled. Compared with OPEN surgery, VATS got the merits of short in-hospital duration (20 days *vs* 27 days, *P*=0.015), less bleeding (197 mL *vs* 250 mL, *P*=0.005) and less pain (4.6 *vs* 6.2, *P*=0.003).

**Conclusion:**

VATS is a safe surgical procedure for early stage NSCLC with merits of lower morbidity and sooner recovery. So it could be concluded that in some circumstances, VATS could be chosen as an alternate of thoractomy.

自20世纪90年代胸腔镜逐步应用于临床以来，胸腔镜肺叶切除术目前已经成为早期非小细胞肺癌（non-small cell lung cancer, NSCLC）治疗的主要手术方式^[1, 2]^。目前国内外已有大量关于胸腔镜手术安全性及有效性的报道^[1-5]^。但对于胸腔镜手术术中意外处理的研究较少。本文对2006年1月-2008年7月在中日友好医院胸外科进行的肺癌手术患者资料进行回顾性研究，旨在对胸腔镜肺叶切除术术中转为胸腔镜辅助或开胸手术及术后相关情况进行分析总结。

## 1 材料与方法

### 1.1 病例及入组标准

收集2006年1月-2008年7月中日友好医院胸外科收治的符合入选标准的NSCLC例。VATS（video assisted thoracoscopy, VATS）入组标准（图 1）包括：NSCLC、根治性肺叶切除/复合肺叶切除术、术前未行新辅助治疗。最终共剔除164例，其中小细胞肺癌29例，开胸探查21例，胸膜固定术37例，冷冻治疗59例，经新辅助治疗18例。最终入组共377例，男性271例，女性106例，平均年龄61岁。根据年龄、性别、术式相匹配原则对同期完成OPEN手术患者进行选择，入组标准同前述，最终共入组129例患者，详细临床资料见表 1。

**1:**
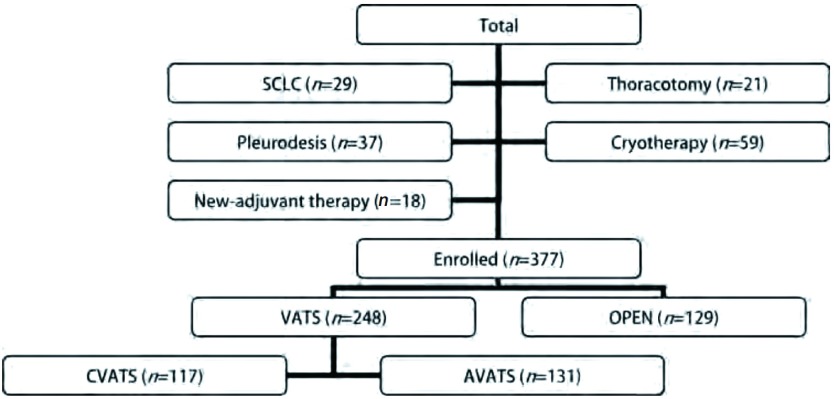
入组筛选过程 Enrolled process. SCLC: small cell lung cancer.

**1:** VATS与OPEN组、CVATS与AVATS亚组临床资料比较 Comparasion of clinical data between VATS and OPEN groups, CVATS and AVATS subgroups

Clinical data	VATS (*n*=248)	OPEN (*n*=129)	*P*	VATS		*P*
				C-VAT (*n*=117)	A-VATS (*n*=131)	
Sex						
Male	166 (66.94%)	105 (81.40%)	0.003	75 (64.10%)	91 (69.47%)	0.471
Female	82 (33.06%)	24(18.60%)		42 (35.90%)	40 (30.53%)	
Age	60	63	0.410	59	61	0.130
Pathology						
AC	76 (30.65%)	39 (30.23%)	0.681	38 (32.48%)	38 (29.01%)	0.554
SCC	85 (34.27%)	66 (51.16%)	<0.001	39 (33.33%)	46 (35.11%)	0.768
Others	87 (35.08%)	24(18.60%)	<0.001	40 (34.19%)	47 (35.88%)	0.781
pTNM Stage						
T1	68 (27.42%)	15(11.63%)	<0.001	41 (35.04%)	27 (20.61%)	0.011
T2	124 (50%)	39 (30.23%)	<0.001	60 (51.28%)	64 (48.85%)	0.703
T3	48 (19.35%)	54 (41.86%)	<0.001	15(12.82%)	33 (25.19%)	0.014
T4	9 (3.63)	21 (16.28%)	<0.001	1 (0.85%)	8(6.11%)	0.027
N0	133 (53.63%)	33 (25.58%)	<0.001	77(65.81%)	56 (42.75%)	<0.001
N1	57 (22.98%)	27 (20.93%)	0.649	22 (18.80%)	35 (26.72%)	0.139
N2	58 (23.39%)	699 (53.49%)	<0.001	18(15.38%)	40 (30.53%)	0.005
AC: adenocarcinoma; SCC: squamous cell cancer.						

### 1.2 手术方法

手术病人均采用双腔气管内插管全身麻醉，单肺通气。完全胸腔镜组（complete video assisted thoracoscopy, CVATS）及胸腔镜辅助组（assisted video as-sisted thoracoscopy, AVATS）全部通过2个切口完成，操作口位于第4或第5肋间腋前线，长约5 cm-7 cm，镜下各肺叶切除操作顺序与传统开胸肺叶切除基本相同。如镜下操作遇淋巴结粘连或转移、出血等情况时，将操作口向肩胛下角方向延长至12 cm-15 cm，沿肋间方向逐层切开皮下组织、背阔肌、前锯肌和肋间肌，放置开胸器牵开肋骨，不切断或切除肋骨，直视下完成肺叶切除和淋巴结清扫，即AVATS。开放手术（open lobectomy, OPEN）采用常规后外侧切口。系统性纵隔淋巴结清扫范围：右侧清扫2、3、4、7、9组淋巴结，左侧清扫2、5、6、7、9组淋巴结。对CVATS中转术式为AVATS或OPEN的患者，最终术式记录为AVATS或OPEN。

### 1.3 观测指标及研究目的

包括术式中转原因、中转机率；不同术式手术时间、术中失血量、术后并发症发生率、术后拔管时间、住院时间、术后引流量、术后早期患者疼痛评分、术后患者肺功能变化等因素。主要目的：评价CVATS中转的为AVATS或OPEN的可能性及常见原因；次要目的：评价VATS的安全性及有效性。肺癌分期采用7^th^版肺癌分期^[6]^。疼痛评分采用视觉模拟评级法^[7]^。

### 1.4 统计学分析

应用SPSS 16.0统计软件进行统计分析，计量资料用Mean± SD显示，组间比较采用独立样本*t*检验；计数资料用率表示，组间比较采用卡方检验。*P* < 0.05为差异有统计学意义。

## 2 结果

2006年1月-2008年7月共实施胸腔镜肺叶切除术248例，其中CVATS 117例，AVATS 131例。在所实施的130例CVATS手术中，CVATS中转为AVATS或OPEN共13例，包括中转为AVATS 9例，中转为OPEN 4例。13例患者中行右肺上叶切除术2例；右肺中叶切除术3例；右肺下叶切除术2例；左肺上叶切除术4例；左肺下叶切除术2例。

中转术式的最常见原因为肺动脉或其分支出血3例（23.08%）和胸腔内粘连3例（23.08%）；其次为血管解剖变异2例（15.39%）、奇静脉出血1例（7.69%）、中叶静脉出血1例（7.69%）；肺门淋巴结无法清除、影响手术解剖过程1例（7.69%）；误伤左主支气管1例（7.69%）；术中冰冻良性，临床怀疑恶性，中转术式进一步取病理1例（7.69%）（表 2）。

**2:** 13例中转术式患者临床资料 Clinical data of the 13 transformed surgical process

No.	Age	Sex	C stage	P stage	Operation	Conversion	Reason
1	64	M	T2aN0M0	T2aN1M0	RML	C to A	Adhesion
2	57	M	T2aN0M0	T2aN0M0	LLL	C to A	Bleeding of superior branch of LLL artery
3	71	F	TlaNOMO	T1bN1M0	LLL	C to A	Obscure pathology
4	66	M	T1bN1M0	T2aN2M0	LUL	C to A	Undisectable of lymph node
5	61	M	T3N0M0	T3N0M0	RML	C to A	Adhesion
6	52	F	T2bN1M0	T2bN1M0	RLL	C to A	Bleeding of inter lobar artery
7	48	M	T3N2M0	T3N2M0	LUL	C to A	Adhesion
8	59	M	T2bN0M0	T2bN1M0	RLL	C to A	Malformation of artery
9	64	F	T1aN1M0	TlaNOMO	RUL	C to A	Bleeding of posterior branch of RUL artery
10	54	M	T2aN1M0	T2aN1M0	LUL	C to O	Left main bronchus injury
11	59	F	T2bN1M0	T2bN2M0	LUL	C to O	Aberrant position of artery
12	63	M	T2aN0M0	T3N2M0	RML	C to O	Injury of RML vein
13	67	M	T1bN1M0	T1bN1M0	RUL	C to O	Injury of azygos vein
F: female; M: male; RML: right middle lobectomy; LLL: left lower lobectomy; LUL: left upper lobectomy; RLL: right lower lobectomy; RUL: right upper lobectomy; C to A: CVATS convert to AVATS; C to O: CVATS convert to OPEN.							

组间比较显示VATS组的住院时间较短（20天 *vs* 27天，*P*=0.015）、术中失血量较少（197 mL *vs* 250 mL, *P*=0.005）。对亚组资料分析显示住院时间、术中失血量、手术时间、并发症发生率等因素均无统计学差异。术后第1天-第3天对所有患者进行疼痛分级，对3天的数据取平均值即为该患者术后早期的疼痛评分。组间比较显示VATS组与OPEN组的疼痛评分分别为4.6±2.1和6.2± 3.4，组间差异具有统计学意义（*P*=0.003）。对亚组数据进行分析显示CVATS组与AVATS组的疼痛评分分别为4.1±2.0和4.9±1.8，组间差异无统计学意义（*P*=0.190）（表 3）。

**3:** VATS与OPEN组、CVATS与AVATS亚组外科数据比较 Comparasion of surgical data between VATS and OPEN groups, CVATS and AVATS subgroups

Surgical data	VATS (*n*=248)	OPEN (*n*=129)	*P*	VATS		*P*
				C-VATS (*n*=117)	A-VATS (*n*=131)	
Complications			0.166			0.614
Yes	23 (9.27%)	18(13.95%)		12(10.26%)	11 (8.40%)	
No	225 (90.73%)	111 (86.05%)		105 (89.74%)	120 (91.60%)	
Fistula (≥7days)	5 (20.16%)	3 (2.33%)		5 (4.27%)	0	
Atrial Fibrillation	3(12.10%)	3 (2.33%)		1 (0.85%)	2(1.53%)	
Pulmonary infection	0	0		0	0	
Empyema	0	0		0	0	
Atelectasis	1 (0.40%)	3 (2.33%)		0	1 (0.76%)	
Fistula	1 (0.40%)	0		0	1 (0.76%)	
Others	13 (5.24%)	9 (6.98%)		6(5.13%)	7 (5.34%)	
Stay (day)	19.57±16.68	26.77±31.16	0.015	17.68±13.74	21.26±18.82	0.087
Time (min)	198.90±63.58	210.58±50.43	0.052	193.80±53.60	203.44±71.22	0.227
Blood loss (mL)	196.50±142.33	250.23±191.28	0.005	191.05±132.78	201.22±150.50	0.575
Extubation (day)	6.75±3.91	6.33±3.87	0.331	7.17±3.88	6.38±3.92	0.116
LN number (*n*)	22.72±9.22	22.35±10.15	0.731	22.30±9.33	23.09±9.15	0.501
N2 station (*n*)	3.17±0.82	3.12±0.32	0.335	3.22±0.59	3.13±0.98	0.363
Drainage (mL)	1, 624±1, 390	1, 468±872	0.200	1, 659±938	1, 589±1, 716	0.695
Pain	4.6±2.1	6.2±3.4	0.003	4.1 ±2.0	4.9±1.8	0.190
pre-FEV_1_(L)	1.95±0.73	2.12±0.99	0.320	2.00±0.91	1.91 ±0.82	0.270
pre-FEV_1_%	(93.26±23.14)%	(91.78±25.62)%	0.450	(95.01 ±18.47)%	(92.58±20.04)%	0.631
post-FEV_1_(L)	1.52±0.38	1.44±0.82	0.002	1.61 ±0.63	1.49±0.40	0.100
post-FEV_1_%	(73.44±18.%)%	(62.59±21.50)%	0.010	(75.34±16.28)%	(70.94±14.60)%	0.187

术前VATS组与OPEN组的一秒通气量（forced expira-tory volume in one second, FEV_1_）、一秒通气量占预计值百分比（forced expiratory volume in one second to forced vital capacity ratio, FEV_1_%）分别为1.95±0.73、（93.26±23.14）%和2.12±0.99、（91.78±25.62）%，组间比较无统计学差异。术后肺功能结果显示CVATS组与OPEN组术后FEV_1_和FEV_1_%均有不同程度的下降，但OPEN组下降更明显，差异具有统计学意义（表 3）。

## 3 讨论

2010年NCCN指南明确指出胸腔镜可以作为无手术禁忌症的NSCLC患者的治疗手段^[8]^。目前胸腔镜已经成为比较成熟的手术方式，但是在一定的情况下仍需要转为开胸手术。其常见原因包括纵隔淋巴结粘连或转移、出血、肿瘤巨大或侵犯纵隔器官、手术器械使用不当、叶间裂分裂差或胸腔粘连等。我院实施的130例CVATS中转的比例为10%，其中9例中转为AVATS，4例中转为OPEN手术，这与Yan^[2]^报道的结果相近。

国内姜冠潮等^[9]^对256例全胸腔镜肺叶切除术患者进行分析认为左肺上叶切除术时中转的几率较大。本组资料显示CVATS术中行左肺上叶切除时中转的机率最高（4/13, 30.77%），行右肺中叶切除时中转的机率次之，其他如右肺上叶、右肺下叶、左肺下叶切除术发生的机率较低。CVATS左肺上叶切除时中转原因有胸腔粘连1例、淋巴结无法清除1例、肺动脉解剖变异1例和误伤左主支气管1例。

Yan等^[2]^报道胸腔镜中转为其他术式最常见的原因依次为手术操作困难、肿瘤体积大、淋巴结粘连、出血、肺裂分化不全等。Nakanishi等^[10]^的研究显示术中肺血管出血是全胸腔镜肺叶切除术中转开胸最常见的原因。本研究数据显示肺动脉或其分支出血和胸腔内粘连是术中最常见的中转原因（3/13, 23.08%），这与Sugi等^[11]^的研究一致。本组因肺动脉出血中转为AVATS 3例，发生于右肺上叶切除术、右肺下叶切除术、左肺下叶切除术各1例，出血动脉分别为右肺上叶后升支动脉、右肺叶间动脉干以及左肺下叶背段动脉。

Doddoli等^[12]^对复发性自发性气胸患者进行VATS手术后分析得出胸腔粘连是VATS手术的相对禁忌症。如胸腔内部粘连或胸腔内肺门血管等部位粘连紧密影响VATS手术操作，有时需中转为AVATS或OPEN手术。本组术中因胸腔内粘连中转3例，其中2例为肺与胸壁广泛粘连，1例为肺门周围组织粘连紧密，无法游离。

淋巴结一般伴随在血管与支气管周围，炎症与结核粘连或肿瘤转移造成淋巴结增大时往往会使局部的解剖结构不清，增加VATS处理血管和支气管的难度。Na-kamura^[13]^认为淋巴结因素在一定程度上影响VATS中转为OPEN的发生率。李运等^[14]^报道172例全胸腔镜手术患者中有9例患者因淋巴结因素需中转术式。本组仅遇到1例术中因淋巴结肿大需要中转术式，其余患者均顺利完成手术，考虑与手术患者的选择有关，另外我们总结术中沿着淋巴结外膜进行仔细分离一般能够将动脉或支气管与淋巴结分离开。

CVATS术中遇到肿瘤体积较小，探查不确实时，可以依据解剖位置进行手术切除。但这种术式肿瘤残留风险较大。如术前在CT引导下对肿瘤进行定位，术中根据定位情况进行切除较准确。但由于术前CT引导下定位可能导致气胸，因此目前在国内应用仍较少。对于体检较小、位置较深的肿瘤一般直接采取AVATS或OPEN术式，本组资料显示由于肿瘤体积小、探查不确实由CVATS中转为AVATS 1例，术中VATS楔形切除组织快速病理未见肿瘤，中转AVATS后取病理确诊为腺癌。

本组中遇到1例术中误操作，行左肺上叶切除术时误切断左主支气管，中转OPEN后行断端切除吻合术，这与胸腔镜经验不足有关。我们总结术中应该常规夹闭目标支气管后膨肺，确定余肺膨胀满意后予以切断，不可盲目切断“目标”支气管。

国外研究^[15-17]^显示与OPEN手术相比，VATS具有损伤小、患者恢复快、住院时间短、术中出血较少、术后并发症发生率较低等优点。本组资料显示与OPEN手术相比，VATS具有住院时间短、术中出血量少的优点。这与其他作者得到的结果类似。但本次研究并未得出VATS在术后拔管时间、术后引流量、术后并发症等方面具有优势，考虑可能与入组病例数较少有关。

术后第1天-第3天对入组患者进行疼痛评分分级，VATS组患者术后早期疼痛较OPEN组轻。这与OPEN组损伤肋骨、肋间神经及肋骨牵开器过度牵拉胸壁引起的损伤等因素有关。

文献^[18]^报道VATS具有减少开胸手术创伤、保护患者肺功能作用。一般认为OPEN手术对术后早期肺功能影响的可能机制包括：疼痛造成限制型呼吸障碍；疼痛导致患者主动咳嗽减少、术后肺泡渗出增多造成阻塞型呼吸障碍；术中切断呼吸肌直接影响呼吸功能。我们考虑术后1周时患者疼痛对呼吸功能的影响较明显，因此在术后4周-5周患者复查时进行肺功能检查，结果显示FEV_1_及FEV_1_%均有不同程度的下降，OPEN组患者下降的幅度较大，考虑组间差异主要是术中直接损伤呼吸肌以及肋间神经造成呼吸肌失神经支配所致。

总之，在严格选择病例进行手术的前提下，胸腔镜手术风险小，可以完成绝大部分早期肺癌及部分中期肺癌患者根治术，并且胸腔镜手术创伤小、患者恢复快、患者术后生活质量较高，因此在一定范围内可以替代OPEN手术。
